# Changes in the New York Schools

**Published:** 1875-05-15

**Authors:** 


### CHANGES IN THE NEW YORK SCHOOLS.

*New York Correspondence of Boston Medical and Surgical Journal, April 29th*, 1875.

By a resolution recently passed by the Commissioners of Charities and Correction, the medical board of Bellevue Hospital has again been changed, and the resolution passed last July rescinded. In order to understand clearly the position of affairs, an account of the old service, and the connection of Bellevue College with the hospital, will be necessary. When Bellevue Hospital Medical College was organized, in 1861, most of the gentlemen of the attending staff, who were not connected with any school, became members of its faculty. This gave the new school a very large representation in the medical board, which it has never lost. In June last, of the eighteen members of the attending staff, Bellevue had ten, the University two, the College of Physicians and Surgeons three, and the outside profession three. In July last, through the influence of three or four medical gentlemen, who were hostile to Bellevue, and were acting in the interest of the University and the Twenty-third Street Medical School, the Commissioners passed a resolution reorganizing the medical board of the hospital, and dividing the service into four equal sections, giving one to each of the colleges and one to the outside profession, making the board to consist of sixteen members. It was also ordered that any vacancy occurring in any division should be filled by and from the class in which the vacancy occurred. This retired six Bellevue men, the loss of whom crippled that school very much, as its course of instruction has always been more clinical, and less didactic, than that of either of the other schools. It was evident that the aggrieved party only wanted the power and the fight would be renewed. This came sooner than any one expected. In January last the old board of commissioners was removed, and a new one appointed by the political party in power. The result has been that the new board, through strictly political influence, has been won over to the Bellevue College interest. To bring about this, the Governor of the State is said to have come down from Albany, with the result of putting back all the old staff, by increasing the medical board to twenty-four members in accordance with a resolution passed March 11th. Immediately on this change being ordered, one of the attending staff, not connected with any college, resigned his position, stating to the commissioners that as they had broken faith with the profession he declined to hold any position in either of their hospitals. It is rumored that changes are to be made in the medical board of Charity Hospital, in the interest of the same school. Already certain political magnates have stated that certain of their friends must go on the board.

There is an unfortunate rivalry among the medical colleges here, which is getting more and more bitter, the end of which no one can foresee ; but it is certain that no real good can result from political wire-pulling, either to the colleges themselves, or to those who have been active in using political influence to bring about very questionable changes.

By the death of Dr. Delafield, the College of Physicians and Surgeons lose their president. The election of Dr. Alonzo Clark to that position would no doubt be very acceptable to the profession, but there is no one to fill his place as professor of theory and practice. The late Dr. Anstie, of London, was offered the place, but he felt compelled to decline on account of family ties. I have heard the names of Dr. Willard Parker and Dr. Buck mentioned in connection with the presidency, but it is only a rumor, and I give it for what it is worth.

The resignation by Dr. F. H. Hamilton of the position of Professor of Surgery in Bellevue College was a surprise to the profession at large. He will be a great loss, as he was considered one of their ablest instructors. The cause of his withdrawal is the same that compelled Dr. Hammond, a year ago, to sever his connection with that college — misunderstanding with the ruling power in the faculty. It is understood that Dr. W. H. Van Buren will fill his place next winter. It is rumored that changes may take place in the other medical schools.

---

INTESTINAL GASES. — Dr. Leven read before a recent meeting of the Society of Biology a communication regarding the gases contained during digestion in the stomach and small intestine. Physiologists have asserted that these gases consist partly of air that has been swallowed with the food, and partly of gases from the blood which have been evolved in the stomach, or in connection with the various chemical processes that accompany the digestive catalysis. Dr. Leven does not, however, share in this opinion, since, as he claims, it precludes the proper investigation of the nature and quantity of intestinal gases.

These gases are always constituted of oxygen, nitrogen and carbonic acid. The nitrogen is constant in its presence, and forms, so to speak, the base. Its most frequent appearance is in the form of a mixture with oxygen, or carbonic acid, yet very often it is found alone, whereas this is never the case with either of the other gases.

Their quantity is almost always invariable whatever may be the food to which the animal may be restricted, whether it is given the most indigestible or the most simple and readily assimilated.

When a dog is given a quantity of fat and cabbage, his stomach and small intestine become reddened, swollen, and eccymotic, but the quantity of gas in the alimentary track is never very considerable in its amount.

Moreover, Dr. Leven denies that there is any relation between the manner of alimentation and the production of gases; the distension and meteorism are caused, he thinks, by a paralysis of the muscular fibres.

To these opinions several members offered objections, but the discussion was deferred until the completion of further experimentation.

Still more recently, it has been announced by M. Bert in the course of these experiments, MM. Planer and Chevreal have discovered the presence of other gases than those named by Dr. Leven, among others, carburets of hydrogen.

At the following meeting, however, Dr. Leven continued his communications upon this subject, by stating that he did not stand alone in having failed to meet with hydrogen and the proto-carburet of hydrogen in the course of his investigations. Other observers than himself had never found them. He also called attention to the fact that if these intestinal gases arise from fermentation of food in the small intestine, these gases should be most abundant during digestion; but the contrary is the case, and it is especially when the animal is fasting that their existence is manifest.—*Progres Medical*, Nos. 7, 8 and 9, 1875. F. J. H.

---

THORACENTESIS IN PNEUMO-THORAX.—The third lecture in the series of clinical lectures, edited by Dr. Seguin, is by Professor Flint, and is entitled "Pneumo-thorax." As there can be no higher authority in all that relates to lung-trouble than the distinguished professor at Bellevue, we quote his remarks on Thoracentesis: "If the pleural cavity become filled after pneumo-thorax has been known to exist, I would not resort to aspiration so long as the quantity of liquid was not large enough to occasion suffering from dyspnœa; that is, assuming that the liquid is not pus, and this is readily ascertained by exploring with the hypodermic syringe; but if there be sufficient dilatation of the chest to occasion dyspnœa, I would withdraw a certain quantity of the liquid, enough to relieve dyspnœa, leaving sufficient to secure the possible advantage of compression; and the withdrawal of liquid within this limit may be repeated *pro re nata*."

Professor Flint gives it as his opinion that the liquid should be withdrawn from the chest always by means of either a canula or catheter. "Puncturing the chest to give exit either to air or air and liquid whenever the suffering from dyspnœa, due to dilatation, is great, is undoubtedly judicious as a merely palliative measure. This I have done repeatedly. But the inquiry has arisen in my mind whether it may not be possible in some rare cases to accomplish something beyond a temporary relief by making a free opening into the chest, as in cases of pneumo-thorax incident to empyema. Let us suppose a case of pneumo-thorax from the bursting of a tuberculous cavity, the amount of phthisis small, the disease non-progressive, and all the circumstances favorable for arrest and recovery, aside from the perforation of lung. There are such cases, albeit they are infrequent. May we not hope that by a free incision, the cure of pneumo-thorax is possible in these cases? The answer to this question must be based on clinical facts which are yet to be acquired. Meanwhile, I can see no objection to making trial of this measure. Pneumo-thorax occurring as a complication of phthisis, is almost hopeless. In the majority of cases, this complication destroys life within a short period. We may say that the prognosis involves only a question of tolerance. It is probable that a free opening into the chest will not shorten the duration of life, and it certainly affords great relief. It is therefore warrantable."—*Am. Practitioner.*

---

THE NATURE OF CHRONIC ALOPECIA.—Dr. J. Pincus (*Berliner Klin. Wochen.*, February 1) says that nineteen out of every twenty cases of baldness are due to the disease known as alopecia furfuracea, or calvities senilis and prematura.

Two views of the pathology of this disease have been held: one ascribing the accompanying atrophy of the cutis to a primary wasting away of the vessels; the other, to an atrophy of the peripheric nerve-fibres. Dr. Pincus believes that a condensation of the connective tissue of the lower layers of the cutis takes place in the earlier stage of the disease, accompanied by a loss of typical length on the part of the hairs, but not in their diameter. At the same time certain alterations take place in the amount and character of the glandular secretion, possibly depending upon the irritation of the condensing connective tissue surrounding them.

In the second stage, the hairs become thin, and this is particularly noticeable in the thickest and woolly hairs, whose roots are situated deeply among the condensing tissue. In the first stage of the affection, the whole head is equally affected, and the hair becomes equally thinned throughout. In the second stage, it is the vertex and forehead which are most severely affected, and while the sides remain stationary, the disease-process goes on in these localities. By the slow and gradual destruction of the nerve-fibres by the condensing connective tissue, the relative sensibility of the skin is much diminished in the affected parts, but without pain.

In the earlier stages of the disease, Dr. Pincus recommends the use of a solution of caustic potassa or soda, one part to two hundred and fifty or five hundred of water, or sodii carbonas, four parts to one hundred to two hundred, or sodii bicarb., four parts to fifty to one hundred and fifty. Of these solutions, two or three tablespoonfuls are to be rubbed into the head for several minutes; at first daily, afterwards oftener. Unfortunately, the prolonged use of this remedy, although of great service, yet has the drawback that it discolors the hair. If it is wished to avoid this, a solution of iodide of potassium, one or two parts to one hundred, will act favorably, though not so rapidly as the former.

In the latter stages, Dr. Pincus uses stronger solutions of these alkalies, with a very weak solution of corrosive sublimate, one part to seven thousand five hundred. He has used all the various irritating and astringent washes, oils, and pomades, without success.—*Phil. Times*, April 24, 1875.

---

INTERNATIONAL MEDICAL CONGRESS.—Arrangements have been made to hold an International Medical Congress early in September, 1876, in Philadelphia, at which discourses will be read upon Medicine and Medical Progress in the United States; on Surgery; Obstetrics; Chemistry and Pharmacy; Materia Medica; Medical Jurisprudence and Toxicology; Hygiene and Social Science; Medical Biography; Medical Education and Institutions; and Medical Literature. The morning sessions will be devoted to general business and reading these discourses, while the afternoon sessions will be devoted to sections on Medicine, on Surgery and Anatomy, on Obstetrics, on Chemistry, Materia Medica, Hygiene, and Medical Jurisprudence, and on Ophthalmology and Otology. This congress will consist of delegates, native and foreign, representing the American Medical Association, the various State medical societies, and the medical societies of Europe, Mexico, the British Dominions, Central and South America, the Sandwich Islands, the East and West Indies, China, and Japan. The congress is to be organized by the election of a president, thirteen vice-presidents, seven of whom shall be natives and six foreigners, a recording secretary, two corresponding secretaries, a treasurer, an executive committee, and a committee on publication. It has been agreed that no vote shall be taken during the sittings of the congress upon any topic discussed or address delivered. The preparation of these discourses has been entrusted to able hands, and it is intended to publish them in an appropriate volume commemorative of the occasion. For the purpose of carrying out these arrangements, a Centennial Medical Commission of Philadelphia has now been fully organized. The president of this commission is Dr. S. D. Gross, and the vice-presidents Drs. Ruschenberger and Stille.—*Boston Med. and Surg. Journal.*

---

A New Way of Operating on the Larynx.—The *Medical Times and Gazette* gives an account of a new method of operating on the larynx, which has been devised by Dr. A. Eysell, of Halle. Every laryngoscopist, he says, must be aware how difficult it is to reach a tumor growing in the lower part of the larynx, which is not movable enough to be driven above the level of the vocal cords by forced expiration. He has, however, succeeded in removing them by the following method: Whilst observing the larynx by means of the laryngoscope, an exceedingly elastic needle is passed through the skin and crico-thyroid membrane, into the larynx, exactly in the median line, and immediately beneath the thyroid cartilage. The needle is then made to transfix the tumor, and by depressing its handle the latter is forced up into the ventricle of the larynx. No hæmorrhage takes place, the only pain felt is during the transfixion of the skin, and no local mischief has followed even frequently repeated operations. If it be intended to cauterize or tear away the tumor, the patient is directed to hold the mirror, or better still, the needle; and in this way Dr. Eysell has succeeded in removing fibromas from the lower part of the laryngeal cavity. He afterwards attempted to operate on tumors, with the needle itself, which could not conveniently be attacked through the mouth, and for this purpose he employed the needle used by Schwartz for performing paracentesis of the tympanum; but even this ought to be gently heated before use, in order to make it more pliable. It was passed, as before, into the larynx, and several incisions or pricks made into the tumor, which was then lifted up and cauterized. In a case where the vocal cords were adherent to one another for their anterior two-thirds, as the result of a suicidal cut throat, which caused considerable shortness of breath on slight exertion, a narrow tenotome was passed through the scar, 0.5 centimetre broad, into the larynx. When the point appeared behind the triangular adhesion, the handle was firmly depressed, and by drawing the knife downward the cords were separated almost to their origins. In the same way, no doubt, injections might be practiced on laryngeal tumors, by the employment of a needle-pointed syringe. It may be impossible to perforate the thyroid cartilage in old people on account of calcification.—*The Doctor.*

---

Heat and Hydrophobia. — The mysterious influence of the "dog-days" upon the canine race, is an opinion of the greatest antiquity, dating back, apparently, to Annubis, the dog form of the Egyptian Apollo, whose appearance in the heavens was a premonition of impending danger. It probably also had some connection with a festival of the Argives, marked by the destruction of many dogs. In the "Iliad," Homer mentions Orion's dog as affecting human health disastrously. Pausanius, in his "Travels in Greece," alluding to the story of Actæon's destruction by his own hounds, was inclined to attribute the myth to the circumstance, that the season had caused the pack of the famous hunter to run mad. Pliny remarks, in his "Historia Naturalis," that canine madness is fatal to man during the heat of Sirius, and proves so in consequence of those who are bitten having a deadly horror of water. For such reason, during the thirty days that this star exerts its influence, we try to prevent the disease by mixing dung from the poultry yard with the dog's food, or else, if he is already attacked with the disease, by giving him hellebore." From the time of Pliny until quite recently, the development of rabies by summer heat has been accepted as a fact among scientific men, and the idea has become too deeply rooted in the popular mind to be easily eradicated. Only within the present century has it been proved conclusively by critical inquiry, that no season of the year is specially concerned in the production of this formidable affection. Hence the absurdity of legislative enactments designed as precautionary measures against hydrophobia, and operative only during the summer months. — DR. C. P. RUSSELL, *in Popular Science Monthly for December.*

---

FORCIPRESSURE.—At a recent meeting of the Societe de Chirurgie of Paris, M. Verneuil called attention to the value of the use of forceps in compressing bleeding vessels, and he gave the name of *forcipressure* to the method, which, however, he did not claim as new. He stated that he was in the habit of applying the forceps in this way when the hæmorrhage was severe, and from a large extent of surface. The instruments might often be left in place for several days.

M. Pean has also made some contributions to the same subject. In a recent communication to the Academy he states that the application of forceps for the arrest of hæmorrhage was indicated in the following three cases:

1. To prevent hæmorrhage, by compressing the vessels before the incisions were made. This procedure was especially useful when peritoneal adhesions were to be cut through.

2. To arrest hæmorrhage temporarily, as in the course of an operation, using the ligature subsequently if the vessels bled.

3. To arrest hæmorrhage definitively; he then applied them, and left them in place with the dressings. They were removed on the first, second, third, or fourth day.

The forceps he used were very much like the ordinary dressing forceps, and the jaws were either T-shaped, diamond-shaped, or triangular. An ordinary rack and pinion attachment was fitted to the handles, and in this way firm compression could be maintained indefinitely. M. Pean had used these forceps in the operation of castration, when he found it desirable to compress the cord. He applied two of them to the cord, removing them on the fourth day.—*Journ. de Med. et de Chir.*, March, 1875.

---

ON NERVES CONTROLLING THE DILATATION OF VESSELS.—Prof. Goltz and others have been making experiments on dogs to determine the effect on the temperature that followed the section of nerves. They found that after dividing the sciatic, the vessels in the affected limb underwent active dilatation, and this result occurred repeatedly in the same limb, if only section were again made of the nerve at a point nearer the periphery. As a result of these experiments, Goltz maintains that on section of a nerve the vaso-dilator fibres contained in it are irritated, and in consequence produce an active dilatation, which, however exhausts itself subsequently, and is followed by a persistent contraction. It is believed further that the tone of the vessels is maintained by certain nerve-centres which exist in the vascular walls. These views are believed to derive support from the fact that they explain certain observed phenomena, as *e.g.*, that the penis of the horse becomes erect after section of the pudic nerve, the vessels of the belly dilate after division of the splanchnic, hyperæmia of the eye follows section of the trigeminus, etc. The author also claims that the condition of irritation, which is constantly observed in the sensitive nerves after section, supports his hypothesis *e.g.*, the pain in the fingers after division of the median nerve.—*Med. Centralbl.*, xii., 41., 1874.—*Schmidt's Jahrb.*

---

Oleate of Mercury (for External Applications).—Much difficulty has been experienced in the preparation of this really valuable mercurial compound. The mistake generally made arises from the erroneous idea that heat is required to effect the union of the constituents. The fact is that the effect of even a gentle heat is detrimental to the preparation, generally causing a reduction of the metal, with the formation of an unsightly grayish-black deposit. Most authorities recommend the use of the yellow oxide of mercury, representing that it is more readily dissolved by the oleic acid than the red oxide. Undoubtedly this is in some sense true, but unless the yellow oxide is sifted into the oleic acid, and well mixed with it by careful stirring, it is apt to aggregate in solid lumps, which resist the action of the solvent a long time. On the other hand, the red oxide, if reduced to a fine powder, dissolves with sufficient rapidity, and requires no special precaution to prevent the formation of lumps.

The following formula in our hands has always yielded a satisfactory product:

℞—
Red Oxide of mercury in fine powder, ʒ vi;
Oleic Acid, purified, Oi.

Mix the oxide with the acid in the cold, and stir occasionally until a transparent solution is obtained. Keep the product in well-stopped bottles, protected from the light.—*Detroit Rev. of Med. and Pharm.*

Terminations of Nerves in Glands.—M. Rouget, at a meeting of the Societe de Biologie, speaking of the different opinions regarding the terminations of nerves in glands, described the venomous glands in the dorsal glands of the larvæ of the salamander as very convenient for the study of that subject. These glands are quite independent of the vessels and form globular groups, into each of which penetrates a nerve that divides into two secondary tubes which arrive at the centre of the glandular cellules, retaining their medullary substance. Thus there is contact between the cellule and the nerve. The presence of a further subdivision cannot be determined; moreover, it is unnecessary to suppose this ulterior subdivision.

M. Rouget has also demonstrated the important fact that in the cockroach there are certain nerves which distribute themselves into cellules and are in contact with the protoplasm of the glandular elements.—*Gaz. Hebdom.* F. J. H.

---

Ergot in Cystic Paralysis.—Dr. J. Willimsky (*Centralblatt fur Chirurgie*) made some experiments with ergot upon the bladders of dogs and rabbits, in which he observed that immediate contraction of that organ ensued, and continued for several seconds. He afterward employed it in the form of injections of the fluid extract in two cases of paralysis with the following results: The first was a marked paralysis occurring in a maiden lady of forty-six; division of a cicatrix between the posterior wall of the bladder and the neck of the uterus proved ineffectual. A small quantity of the fluid extract was then injected daily into the previously cleansed bladder, and a complete cure was effected in fourteen days. The second was a case of acute cystic catarrh with inability to completely empty the bladder. Three injections at intervals of twenty-four hours afforded entire relief.—*The Clinic*, May, 1875.

M. PERRINE stated, at a recent meeting of the Surgical Society of Paris, that the method of administering ether by means of a cone covering the mouth and nose, was invented by M. Bonnet, of Lyons! Does he know who first gave it on a towel, and who first on a napkin?

And where is the "Dr. Colton of the United States" who is reported to the same august body as having administered nitrous oxide in 67,000 cases? F. J. H.

AN ANTIDOTE TO CHLOROFORM—Dr. Schueller has discovered that the nitrite of amyl quickly removes the effects of chloroform on the vessels of the pia mater, and that even in cases of advanced narcotism from the latter drug it rapidly relieves the dyspnœa and labored respiration, restoring the strength of the pulse and the reflex excitability. This discovery may prove of much practical value where chloroform continues to be the favorite anæsthetic.—*Rich. and Lou. Journal.*

TREATMENT OF PEMPHIGUS.—Dr. Hillariet, founding his plan of treatment upon the analogy which exists between pemphigus and the second degree of burns, employs in cases of this affection, which is generally so rebellious to treatment, the remedies found useful in burns, viz., the covering of the parts with wadding soaked in oleo-calcareous liniment. The pruritus soon abates, and the bullous eruption ceases at the end of a variable period.—*Union Medicale*—*Med. Times and Gazette*, April 3, 1875.

THE treatment of jaundice by electricity, has often proved successful. The procedure is certainly worthy of trial. The position of the gall-bladder is first found out by percussion, and to this point the electrode of an inductive electric machine is applied. The other electrode is placed on the opposite side of the abdominal wall. When the current is passed, a gurgling sound may be heard, and the cure is often immediate.—*Am. Med. Weekly.*

